# Impact of Prematurity and Perinatal Antibiotics on the Developing Intestinal Microbiota: A Functional Inference Study

**DOI:** 10.3390/ijms17050649

**Published:** 2016-04-29

**Authors:** Silvia Arboleya, Borja Sánchez, Gonzalo Solís, Nuria Fernández, Marta Suárez, Ana M. Hernández-Barranco, Christian Milani, Abelardo Margolles, Clara G. de los Reyes-Gavilán, Marco Ventura, Miguel Gueimonde

**Affiliations:** 1 Department of Microbiology and Biochemistry of Dairy Products, Instituto de Productos Lácteos de Asturias (IPLA-CSIC), 33300 Villaviciosa, Asturias, Spain; silvia.arboleya@teagasc.ie (S.A.); borja.sanchez@csic.es (B.S.); ahb@ipla.csic.es (A.M.H.-B.); amargolles@ipla.csic.es (A.M.); greyes_gavilan@ipla.csic.es (C.G.d.l.R.-G.); 2 Pediatrics Service, Hospital Universitario Central de Asturias, SESPA, 33006 Oviedo, Asturias, Spain; GSOLIS@telefonica.net (G.S.); msr1070@hotmail.com (M.S.); 3 Pediatrics Service, Hospital de Cabueñes, SESPA, 33394 Gijón, Asturias, Spain; nuriajmhd@gmail.com; 4 Laboratory of Probiogenomics, Department of Life Sciences, University of Parma, 43124 Parma, Italy; christian.milani@genprobio.com (C.M.); marco.ventura@unipr.it (M.V.)

**Keywords:** intestinal microbiota, microbiome, preterm, infants, antibiotics

## Abstract

Background: The microbial colonization of the neonatal gut provides a critical stimulus for normal maturation and development. This process of early microbiota establishment, known to be affected by several factors, constitutes an important determinant for later health. Methods: We studied the establishment of the microbiota in preterm and full-term infants and the impact of perinatal antibiotics upon this process in premature babies. To this end, 16S rRNA gene sequence-based microbiota assessment was performed at phylum level and functional inference analyses were conducted. Moreover, the levels of the main intestinal microbial metabolites, the short-chain fatty acids (SCFA) acetate, propionate and butyrate, were measured by Gas-Chromatography Flame ionization/Mass spectrometry detection. Results: Prematurity affects microbiota composition at phylum level, leading to increases of Proteobacteria and reduction of other intestinal microorganisms. Perinatal antibiotic use further affected the microbiota of the preterm infant. These changes involved a concomitant alteration in the levels of intestinal SCFA. Moreover, functional inference analyses allowed for identifying metabolic pathways potentially affected by prematurity and perinatal antibiotics use. Conclusion: A deficiency or delay in the establishment of normal microbiota function seems to be present in preterm infants. Perinatal antibiotic use, such as intrapartum prophylaxis, affected the early life microbiota establishment in preterm newborns, which may have consequences for later health.

## 1. Introduction

Increasing knowledge about the important role the intestinal microbiota play in human health has attracted the attention of researchers to factors that determine its development in the host. During the last decade the improvement and availability of next-generation DNA sequencing techniques has allowed for carrying out detailed gut microbiota studies, increasing enormously our understanding of its composition and activity [1,2,3,4,5,6,7]. The microbiota varies along the gastrointestinal tract, corresponding with different ecological niches from the mouth to the colon. The upper bowel is sparsely populated, while the colon is heavily colonized, with levels that may reach 10^11^–10^12^ bacteria per gram of content [8].

The key role of early life events in determining the microbiota establishment process in the newborn, and their impact on later health, is currently well recognized [9,10]. This early colonization is essential for adequate infant development, constituting the basis for the later physiological, immunological, and neurological homeostasis of the individual [11,12,13,14].

The process of establishment of the microbiota starts at birth and is driven by the interplay between genetic factors, mode of delivery, environment, and feeding mode and later diet [15]. Recent studies have monitored the establishment and development of the microbiota in the first days of infant life [16,17], increasing our understanding of the step-wise evolution of this process. Fecal metabolome studies have also been carried out underlining the strong relationship between the development of the microbiota and that of the metabolic pathways in the infant gut [17]. During the very early days the infant fecal microbiota seems to rely mainly in the catabolism of proteins with production of free amino acids and branched chain fatty acids, with high levels of proteobacteria. Then the infant microbiota turns towards the metabolism of carbohydrates with a concomitant increase in the production of short-chain fatty acids (SCFA) [17]. This initial intestinal colonization process, in spite of being a critical moment for microbiota development and modulation, remains poorly understood with significant gaps in the knowledge about the factors affecting it, perhaps with the exception of the mode of delivery and feeding patterns [16,18,19]. In spite of the existing gaps, a recent study has started to take advantage of this knowledge and demonstrated the beneficial effect, in terms of microbiota modulation, of vaginal microbiota transfer in those cases in which the microbiota establishment process may be challenged, as is the case in C-section delivered babies [20].

Prematurity is known to affect different systems; these infants are born with an immature immune system [21], which limits their resistance to infection, thereby increasing their risk of disease. In a previous work [22] we studied the process of establishment of the intestinal microbiota in very-low birth-weight (VLBW) preterm infants and compared it with that of healthy full-term, vaginally delivered, exclusively breast-fed (FTVDBF) neonates by 16S rRNA gene profiling and quantitative PCR for different microbial groups. The results obtained showed clear differences in the process of intestinal microbiota development in VLBW preterm infants, which is in good agreement with previous observations [23,24,25,26,27,28]. In addition, we found that perinatal antibiotic use, including intrapartum antimicrobial prophylaxis (IAP), affects the gut microbiota development during the critical first weeks of life. These results have been further confirmed by more recent studies [29] underlining the importance of IAP as a factor influencing the microbiota establishment in the newborn.

In the present work, we analyzed the data from our previous study [22] at phylum levels and further extend our study by conducting a functional inference analysis to determine the potential impact of prematurity and perinatal antibiotics upon the genes present in the intestinal microbiota. Moreover, we determined the levels of the main intestinal bacterial metabolites, the fecal SCFA acetate, propionate and butyrate, and compared them among the infant groups.

## 2. Results and Discussion

### 2.1. Establishment of the Intestinal Microbiota in Preterm and Full-Term Infants

**Figure 1:**
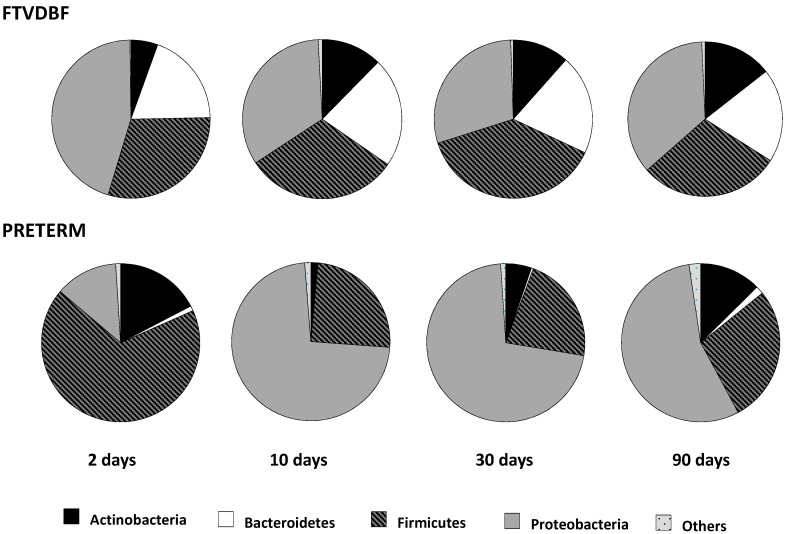
Aggregate microbiota (%) at phylum level in fecal samples from full-term, vaginally delivered, exclusively breast-fed (FTVDBF) *vs.* preterm infants at the different time points analyzed.

The analyses of the bacterial phyla present in fecal samples from VLBW preterm and FTVDBF babies evidenced clear differences between both groups of infants during the first months of life (Figure 1). Preterm neonates harbored a significantly higher (*p* < 0.01) relative proportion of Firmicutes at two days of age, and of Proteobacteria in the later sampling times (*p* < 0.01 at 10 and *p* < 0.001 at 30 days of age), than FTVDBF babies. By contrast, premature infants showed reduced levels (*p* < 0.05) of Bacteroidetes at day 2 of life and of this phylum and that of Actinobacteria during the first month, the differences remaining significant (*p* < 0.05) for up to three months in the case of the phylum Bacteroidetes. In FTVDBF babies Proteobacteria, followed by Firmicutes and Bacteroidetes, dominated the microbiota at two days of age, while in preterms Firmicutes followed by Actinobacteria and Proteobacteria were the predominant groups (Figure 1). At 10 days of age in FTVDBF newborns Proteobacteria and Firmicutes co-dominate with a slight increase in the percentage of sequences from Bacteroidetes and Actinobacteria, a situation that remained stable with only minor changes during the rest of the study. However, at the same age (10 days) Proteobacteria had become the clearly dominant population in VLBW preterm infants, followed by Firmicutes, Actinobacteria, and Bacteroidetes. This situation remained unchanged during the first month of life, with the levels of Firmicutes and Actinobacteria increasing later on (at 90 days) (Figure 1).

Previous studies reported the impact of prematurity upon the process of development of the intestinal microbiota in the neonate [25,27,30,31,32,33]. Our results on the bacterial phyla present in the samples, which confirm our previous data, show noticeable differences in the gut microbiota composition between preterm and FTVDBF babies [22,31].

**Figure 2:**
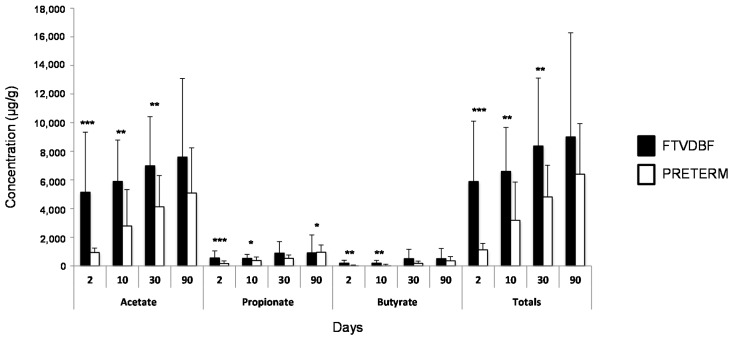
Concentration (mean ± SD) of the main SCFAs (acetate, propionate, and butyrate) and total SCFAs in fecal samples from full-term, vaginally delivered, exclusively breast-fed (FTVDBF) *vs.* preterm infants at the different time points analyzed (2, 10, 30, and 90 days). Asterisks indicate statistically significant differences (* *p* < 0.05; ** *p* < 0.01, *** *p* < 0.001).

Despite the high inter-individual variation, and in accordance with the observed alterations on the fecal microbiota composition, differences in the levels of SCFA between VLBW preterm and FTVDBF infants were also observed. As expected, acetate was the major SCFA in both groups of infants, followed by propionate and low levels of butyrate. We found a significantly (*p* < 0.05) lower concentration of total fecal SCFA in our low-birthweight preterm infants when compared with the FTVDBF ones. These differences were evident at the first sampling points but tended to disappear along the study period (Figure 2). These results are in good agreement with previously reported data obtained in non-low-birthweight premature babies [25]. The alteration in the SCFA pattern in premature babies indicates a strong metabolic effect of the observed differences in microbiota composition, suggesting an important alteration of the intestinal microbiota’s functionalities.

In this regard, when the results of the functional inference analyses of both groups of infants (VLBW preterm and FTVDBF) were compared, we found differences between them. By collapsing the data at different KEGG levels we found the KEGG level 1 categories Metabolism, Cellular Processes, Environmental Information Processing, and Genetic Information Processing to be the most affected, with most of the pathways at KEGG level 2 also displaying statistically significant differences (*p* < 0.05 and *q* < 0.25) between preterm and FTVDBF infants (Table 1). Metabolism was the most affected category, with preterm infants showing a significantly (*p* < 0.05 and *q* < 0.25) higher frequency of genes from the pathway “xenobiotics biodegradation and metabolism” along the whole study period, but without reaching statistically significant differences at 90 days of life. Similarly, preterm babies displayed significantly higher frequencies of genes from the pathways “lipid metabolism” at two days of life and a trend toward higher levels at 10 days, “metabolism of other amino acids”, “metabolism of terpenoids and polyketides”, and “nucleotide metabolism” at two days of age (Table 1). On the other hand, preterm babies showed lower numbers of genes belonging to other pathways such as “energy metabolism”, “enzyme families”, “glycan biosynthesis and metabolism”, “metabolism of cofactors and vitamins”, “biosynthesis of other secondary metabolites”, “carbohydrate metabolism”, or “amino acid metabolism”.

Regarding the categories Cellular Processes, Genetic Information Processing, and Environmental Information Processing, preterm infants showed a higher relative frequency (*p* < 0.05 and *q* < 0.25) than FTVDBF infants of inferred genes belonging to the pathways “cell growth and death” and “signaling molecules and interaction” at two days of age, while the opposite was observed for the later sampling points (Table 1). A similar behavior was observed for inferred genes belonging to the “translation” and “replication and repair” pathways. The opposite behavior—lower levels at two days in preterm infants but higher later on—was observed for the “transcription”, “cell motility”, and “signal transduction” pathways. Moreover, in general, lower frequencies of inferred genes from the pathways “transport and catabolism” and “folding, sorting, and degradation” were observed in preterm than in full-term infants during the study period, the contrary being true for “membrane transport” (Table 1).

The functional inference analysis carried out suggests an increasing ability of the preterm infant microbiota to metabolize xenobiotics and a trend toward increased presence of genes related with cell motility, likely related to the dominance of Proteobacteria in this infant group. Moreover, a concomitant reduction in the presence of common intestinal microbiota metabolic activities such as glycan metabolism, or metabolism of vitamins, was observed in VLBW preterm infants when compared with FTVDBF babies, suggesting a reduced presence of genes related to the functions of the normal microbiota. In general, as was observed for the microbial composition data, the differences tended to be reduced over time, in most cases having disappeared by the age of 90 days (Table 1). A good correlation between total metagenomics and functional inference from 16S rDNA gene profiling has been previously reported [34], underlining the interest of performing functional inference analyses when such data are available. It is important to underline that at the end of the study none of the preterm infants had been exclusively breast-fed or formula-fed and the exposure to breastmilk was highly variable, as is often the case among preterm babies, while our control group included only exclusively breast-fed babies. Therefore, the potential impact of the different feeding habits cannot be overruled as a factor explaining the differences observed between both groups of infants.

In accordance with previous studies [22,25,31], our results suggest a deficiency or delay in the establishment of a normal microbiota function in the preterm infant gut. Although this deficiency seems to have been mostly overcome by the age of three months, given the important role of the early days of life for the maturation of the immune system [21], it could be hypothesized that this alteration in the early colonization may pose a risk for later health.

### 2.2. Effect of Perinatal Antibiotics on Microbiota Development in Preterm Infants

**Figure 3:**
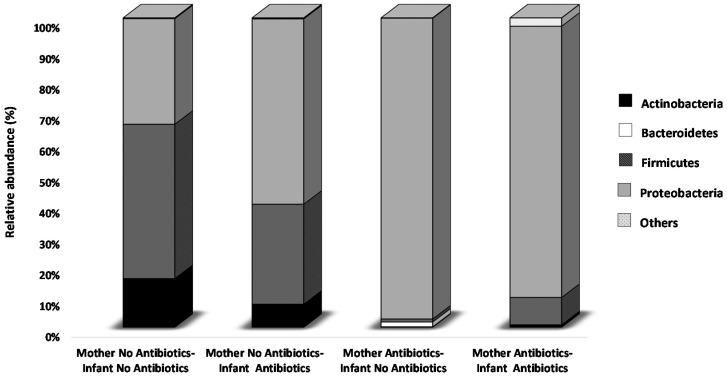
Relative abundance (%) of phylum level distributions of the fecal microbiota in the different antibiotic exposure groups classified into four classes depending on mother and infant antibiotic exposure at 30 days of life.

In this study we compared four groups of VLBW preterm infants established on the basis of the administration of antibiotics to the mother (Intrapartum antimicrobial prophylaxis, IAP), to the infant itself, to both mother and infant, or to none of them (no antibiotic-exposed infants). The results obtained confirmed, at a high taxonomic level, the previously reported effect of perinatal antibiotics (including IAP) upon the preterm newborn microbiota [22]. Now the comparison of the 16S rRNA profiling data at phylum level, among the different antibiotic exposure groups, allowed us to identify antibiotic-related effects upon the microbiota composition. Interestingly, these effects were not so apparent in the first days of life, when no statistically significant differences on the bacterial phyla were observed among the four preterm infant groups (data not shown), as after 30 days when statistically significant differences were found for Actinobacteria (*p* < 0.05), Firmicutes (*p* < 0.01), and Proteobacteria (*p* < 0.01) (Figure 3). The relative frequency of the first phylum was significantly higher in antibiotic-free preterm infants than in the groups where either the mother or the mother and the infant received antibiotics (*p* < 0.01 and *p* < 0.05, respectively). The phylum Firmicutes showed significantly higher levels in antibiotic-free babies with respect to the two infant groups where mothers received antibiotics (*p* < 0.01 in both cases). Interestingly, this phylum was also found to be higher (*p* < 0.05) in the antibiotic-receiving preterm babies whose mothers did not receive IAP than in the antibiotic-free preterm babies whose mothers were administered antibiotics. The opposite behavior to that found for Firmicutes was observed for the phylum Proteobacteria, whose levels were lower in antibiotic-free newborns than in those cases in which either the mother (*p* < 0.05) or the mother and the infant (*p* < 0.01) received antibiotics. Moreover, higher Proteobacteria levels (*p* < 0.05) were found in infants whose mothers received IAP than in the group in which the infants but not the mothers received the antibiotics (Figure 3).

Our results support and extend previous data indicating an effect of perinatal exposure to antibiotics on the establishing microbiota. Increases in family *Enterobacteriaceae* (microorganisms belonging to the phylum Proteobacteria) following antibiotic exposure in neonates have been repeatedly observed [22,29,35,36,37]. Although some authors did not find any effect of maternal antibiotics use during pregnancy on the microbiota development in full-term infants [38], other studies did [36]. Moreover, in the specific case of IAP, microbiota alterations such as a reduction in the levels of the family *Bacteroidaceae* have been observed in both preterm and full-term infants [22,29,39]. Notably, the observed effect of antibiotics was more pronounced when the antibiotics were administered to the mother during delivery (IAP) that when the infant itself received them. Moreover, it was also interesting to note that this alteration on the microbiota development process was not so evident during the first days of life as at the end of the first month, having almost disappeared by the age of three months.

In accordance with the microbial data, the concentration of SCFA was also found to be affected, especially by the IAP administration to the mothers. The high inter-individual variability on SCFA levels is likely to have prevented the detection of any statistically significant difference among the four groups of infants. Nevertheless, at 30 days of age infants not exposed to antibiotics at all, or those who received them but whose mothers did not, showed a trend towards higher levels of acetic (*p* = 0.075) and total (*p* = 0.060) SCFA than the newborns whose mothers received IAP (Table S1).

**Figure 4:**
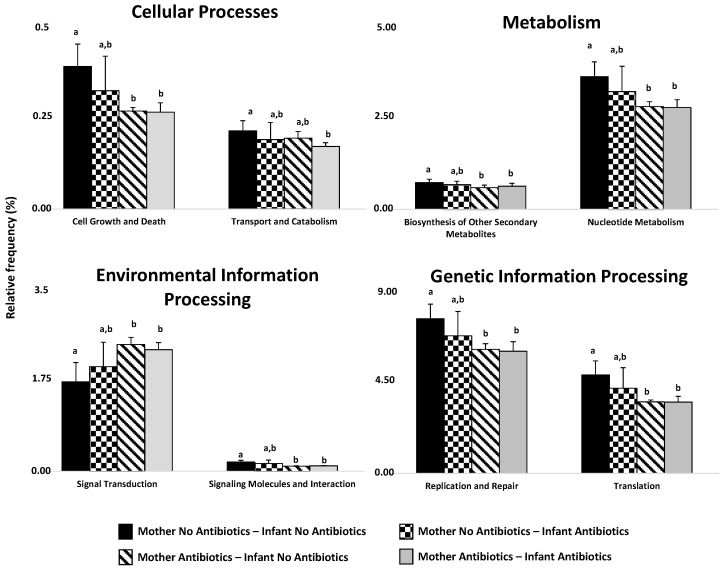
Relative frequencies (mean ± SD) of KEGG pathways (level 2) belonging to the categories that showed statistically significant differences among the four different antibiotic exposure groups at 30 days of life. Different letters above columns within the same KEGG category indicate statistically significant differences (*p* < 0.05 and *q* < 0.25).

The results of the functional inference analyses showed differences among the four different antibiotic exposure groups. In accordance to the microbiota composition data, the age of 30 days was the time at which statistically significant differences (*p* < 0.05 and *q* < 0.25) among the groups were observed for more functional pathways (Figure 4). The categories Cellular Processes, Metabolism, Environmental Information Processing, and Genetic Information Processing, were the most affected. Interestingly, the administration of IAP to the mothers was the most influential factor. The relative frequency of inferred genes belonging to the pathways “cell growth and death,” “biosynthesis of other secondary metabolites,” “nucleotide metabolism,” “signaling molecules and interaction,” “replication and repair,” and “translation” were significantly higher (*p* < 0.05 and *q* < 0.25) in the antibiotic-free babies group with respect to the two groups of babies whose mothers received IAP. Interestingly, no differences were evident for the abovementioned pathways between the groups of antibiotics-free babies and the antibiotic-receiving preterm newborns whose mothers did not receive IAP. The opposite behavior was observed for the relative frequency of inferred genes belonging to “signal transduction” (Figure 4), for which higher levels were observed in the infant groups whose mothers received IAP than in babies not exposed to antibiotics. The functional inference analyses carried out suggest pathways potentially affected by the use of perinatal antibiotics, mainly of IAP administration to the pregnant woman.

These results indicate an effect of perinatal antibiotics, mainly of IAP, on early life microbiota, which may involve a lasting effect on the individual physiology [13]. The use of antibiotics may influence microbiota-host crosstalk during the neonatal period, which may have profound consequences for later health [15]. Actually, perinatal antibiotics exposure has been reported to increase the risk of later disease such as asthma [40,41]. Given that the estimated use of IAP is over 30% of total deliveries [42], greater attention should be paid to its potential impact upon the gut microbiota. This impact should be considered as a factor in the decision on whether or not to administer IAP. In general, intrapartum prophylaxis is recommended in the case of premature rupture of membranes and, in some countries in which a screening protocol is established, when vaginal group B streptococci colonization is observed [43,44]. However, there are other cases in which antibiotics are administered without a clear benefit [45,46] and in which the use of IAP would be arguable, especially since we are starting to understand their impact on the early microbiota development and the importance of this process for later health [7,9,47]. It would be advisable to develop strategies, such as the concomitant administration of probiotics, aimed at limiting the impact of IAP in the establishing microbiota in those cases in which IAP is required.

## 3. Materials and Methods

### 3.1. Volunteers and Samples

Thirteen Caucasian FTVDBF infants and 27 Caucasian very low birthweight (VLBW) preterm infants from a previous work [22] were included in this study. Fourteen of the preterm infants’ mothers received intrapartum antimicrobial prophylaxis (IAP) (penicillin, ampicillin, or ampicillin plus erythromycin). Twelve infants received antibiotics already during the first week of life, while five additional infants started antibiotic treatment during the second week. Only five out of the 27 mother/premature infant pairs were not exposed to antibiotics, either intrapartum or postnatally, and in nine of the pairs the mother received IAP and the infant postnatal antibiotics. Fecal samples were collected at the hospital between 24 and 48 h of life and at 10, 30, and 90 days of age, immediately frozen at −20 °C and processed as described by Arboleya and co-workers [22].

### 3.2. Intestinal Microbiota Analyses

The data from the abovementioned previous study [22], deposited at the National Center for Biotechnology Information (ShortRead Archive BioProject ID PRJNA230470), were now used to assess microbial composition at high phylogenetic (phylum) level. These data were obtained from sequencing of amplicons covering the V3 region generated with optimized primers [48]. UCLUST software and the Ribosomal Database Project were used for phylogenetic assignations.

### 3.3. Determination of SCFA in Feces

The analysis of short chain fatty acids (SCFA) was carried out in a chromatographic system composed of two 6890N GC (Agilent Technologies Inc., Palo Alto, CA, USA) connected to a FID and a MS 5973N detector as described previously [25].

### 3.4. Functional Inference Analysis

The functionality of the different metagenomes was predicted using the software PICRUSt 1.0.0 (http://picrust.github.com) [49]. In short, this software allows the prediction of functional KEGG pathways abundances from the 16S rDNA reads. Firstly, a collection of closed reference OTUs was obtained from the filtered reads using QIIME v1.7.0 [50] by querying the data against the IMG/GG reference collection (version 13.5, May 2013, http://greengenes.secondgenome.com). Reverse strand matching was enabled during the query and OTUs were picked at a 97% identity. A BIOM-formatted table [51] was obtained with the pick_closed_reference_otus.py script. This table, containing the relative abundances of the different reference OTUs in all the metagenomes, was normalized by the predicted 16S rDNA copy number with the script normalize_by_copy_number.py. Final functional predictions, inferred from the metagenomes, were created with the script predict_metagenomes.py. When necessary, tab-delimited tables were obtained with the script convert_biom.py.

Predicted metagenomic contents were collapsed at two hierarchical KEGG pathway levels (levels 1 and 2) (http://www.genome.jp/kegg/pathway.html) with the categorize_by_function.py script. Each of these tables was analyzed statistically in STAMP v2.0.0 [52]. Data of the KEGG pathway distributions, at different hierarchical levels, were plotted with the script summarize_taxa_through_plots.py.

### 3.5. Statistical Analyses

In the functional inference analyses, the association of KEGG pathways at the different hierarchical levels with the different grouping variables was identified by the two-sided Welch’s test for the analysis of preterm and full-term grouping variables; Kruskal–Wallis was used for the analysis of the four antibiotics grouping variables; and White´s non parametric *t*-test was used on every pairwise antibiotic group comparison. The correction FDR [53] was finally applied in all cases and significant differences in KEGG pathways between infant groups were only considered below a *p*-value of 0.05 and a *q*-value below 0.25 [34]. With regard to the microbiota composition and SCFA results were analyzed using SPSS software (SPSS Inc., Chicago, IL, USA). The differences among infant groups were analyzed using the non-parametric Kruskal–Wallis test or, in the case of pairwise comparison, the Mann–Whitney *U*-test. Statistical significance was accepted at *p* < 0.05.

## 4. Conclusions

Our results indicate a delay in the establishment of the normal microbiota function in preterm infants. Moreover, these early microbiota alterations are further affected by the use of perinatal antibiotic, such as intrapartum prophylaxis, in these infants. This alteration in the early microbiota establishment may have profound consequences for later health. Therefore, it would be advisable to develop strategies aimed at limiting the impact of prematurity and antibiotics use upon the establishing microbiota.

## Figures and Tables

**Table 1:** Relative frequencies (mean ± standard deviation (SD)) at the different time points of Kyoto Encyclopedia of Genes and Genomes (KEGG) pathways (level 2) belonging to the categories that showed statistically significant differences between preterm and full-term infants. Asterisk denotes statistically significant differences (*p* < 0.05 and *q* < 0.25) after Fañse Discovery Rate (FDR) correction; ns: Non-statistically significant differences.

KEGG Pathways	Infants	Day 2			Day 10			Day 30			Day 90		
		Mean ± SD	*p* (*t*-Test)	*q*-Value	Mean ± SD	*p* (*t*-Test)	*q*-Value	Mean ± SD	*p* (*t*-Test)	*q*-Value	Mean ± SD	*p* (*t*-Test)	*q*-Value
**Metabolism**													
**Amino Acid Metabolism**	**Preterm**	8.68 ± 0.58	ns	0.558	8.29 ± 0.64	*	0.031	8.35 ± 0.48	*	0.053	8.61 ± 0.66	ns	0.263
	**Term**	8.43 ± 1.02			9.06 ± 0.78			8.86 ± 0.61			9.00 ± 0.67		
**Biosynthesis of Other Secondary Metabolites**	**Preterm**	0.77 ± 0.06	ns	0.776	0.66 ± 0.08	*	0.037	0.70 ± 0.10	*	0.056	0.75 ± 0.11	ns	0.391
	**Term**	0.74 ± 0.24			0.86 ± 0.24			0.85 ± 0.19			0.82 ± 0.22		
**Carbohydrate Metabolism**	**Preterm**	11.53 ± 0.64	ns	0.383	10.99 ± 0.77	ns	0.902	10.86 ± 0.7	*	0.131	10.88 ± 0.60	ns	0.497
	**Term**	11.16 ± 1.08			11.04 ± 0.80			11.31 ± 0.73			11.11 ± 0.76		
**Energy Metabolism**	**Preterm**	4.93 ± 0.19	ns	0.544	4.72 ± 0.12	*	0.033	4.75 ± 0.13	*	0.029	4.91 ± 0.31	*	0.550
	**Term**	5.08 ± 0.63			5.2 ± 0.52			5.13 ± 0.42			5.26 ± 0.46		
**Enzyme Families**	**Preterm**	2.09 ± 0.07	ns	0.809	2.03 ± 0.05	*	0.039	2.03 ± 0.04	*	0.023	2.09 ± 0.07	ns	0.327
	**Term**	2.08 ± 0.15			2.13 ± 0.10			2.13 ± 0.09			2.13 ± 0.09		
**Glycan Biosynthesis and Metabolism**	**Preterm**	1.89 ± 0.28	*	0.027	2.25 ± 0.38	*	0.112	2.35 ± 0.25	ns	0.307	2.22 ± 0.31	ns	0.343
	**Term**	2.73 ± 0.82			2.71 ± 0.78			2.60 ± 0.67			2.50 ± 0.73		
**Lipid Metabolism**	**Preterm**	3.08 ± 0.24	*	0.006	2.89 ± 0.30	ns	0.493	2.78 ± 0.12	ns	0.561	2.85 ± 0.17	ns	0.640
	**Term**	2.83 ± 0.16			2.81 ± 0.22			2.82 ± 0.19			2.90 ± 0.22		
**Metabolism of Cofactors and Vitamins**	**Preterm**	3.55 ± 0.55	*	0.044	3.80 ± 0.19	*	0.032	3.88 ± 0.26	ns	0.291	3.89 ± 0.13	ns	0.387
	**Term**	4.07 ± 0.52			4.14 ± 0.39			4.02 ± 0.34			4.03 ± 0.18		
**Metabolism of Other Amino Acids**	**Preterm**	1.66 ± 0.06	*	0.230	1.68 ± 0.08	ns	0.892	1.70 ± 0.06	ns	0.367	1.65 ± 0.13	ns	0.510
	**Term**	1.63 ± 0.07			1.69 ± 0.13			1.68 ± 0.07			1.61 ± 0.14		
**Metabolism of Terpenoids and Polyketides**	**Preterm**	1.74 ± 0.13	*	0.052	1.50 ± 0.19	ns	0.620	1.44 ± 0.08	*	0.034	1.46 ± 0.08	ns	0.295
	**Term**	1.57 ± 0.20			1.53 ± 0.14			1.53 ± 0.09			1.52 ± 0.12		
**Nucleotide Metabolism**	**Preterm**	4.27 ± 0.52	*	0.125	3.23 ± 0.63	*	0.030	3.27 ± 0.57	*	0.064	3.40 ± 0.46	ns	0.279
	**Term**	3.85 ± 0.63			3.78 ± 0.57			3.70 ± 0.51			3.67 ± 0.40		
**Xenobiotics Biodegradation and Metabolism**	**Preterm**	2.57 ± 0.35	*	0.000	2.23 ± 0.35	*	0.082	2.18 ± 0.28	*	0.036	2.08 ± 0.41	ns	0.269
	**Term**	1.80 ± 0.15			1.84 ± 0.35			1.87 ± 0.31			1.80 ± 0.39		
**Cellular Processes**													
**Cell Growth and Death**	**Preterm**	0.50 ± 0.09	*	0.064	0.30 ± 0.09	*	0.034	0.30 ± 0.08	*	0.027	0.35 ± 0.10	ns	0.289
	**Term**	0.40 ± 0.11			0.40 ± 0.10			0.40 ± 0.08			0.41 ± 0.08		
**Cell Motility**	**Preterm**	1.12 ± 0.47	*	0.151	2.06 ± 1.10	ns	0.496	1.98 ± 0.97	*	0.121	1.84 ± 0.81	ns	0.930
	**Term**	1.85 ± 1.31			1.67 ± 1.30			1.51 ± 0.59			1.78 ± 0.91		
**Transport and Catabolism**	**Preterm**	0.20 ± 0.03	*	0.200	0.18 ± 0.03	*	0.049	0.19 ± 0.03	*	0.085	0.20 ± 0.06	ns	0.317
	**Term**	0.29 ± 0.18			0.32 ± 0.19			0.29 ± 0.17			0.31 ± 0.18		
**Genetic Information Processing**													
**Folding, Sorting and Degradation**	**Preterm**	2.20 ± 0.05	*	0.100	2.18 ± 0.14	*	0.120	2.19 ± 0.10	*	0.112	2.20 ± 0.11	ns	0.749
	**Term**	2.40 ± 0.28			2.34 ± 0.28			2.31 ± 0.20			2.32 ± 0.14		
**Replication and Repair**	**Preterm**	8.76 ± 1.06	*	0.126	6.52 ± 1.21	*	0.046	6.50 ± 0.98	*	0.035	7.00 ± 1.14	ns	0.360
	**Term**	7.91 ± 1.29			7.88 ± 1.30			7.60 ± 1.07			7.76 ± 0.99		
**Transcription**	**Preterm**	2.70 ± 0.19	ns	0.839	3.02 ± 0.18	*	0.029	3.00 ± 0.19	*	0.191	3.05 ± 0.20	ns	0.261
	**Term**	2.72 ± 0.29			2.73 ± 0.31			2.86 ± 0.27			2.90 ± 0.26		
**Translation**	**Preterm**	5.70 ± 0.82	*	0.053	3.93 ± 0.99	*	0.039	3.93 ± 0.82	*	0.045	4.19 ± 0.73	ns	0.266
	**Term**	4.74 ± 1.08			4.81 ± 0.99			4.68 ± 0.80			4.57 ± 0.58		
**Environmental Information Processing**													
**Membrane Transport**	**Preterm**	15.15 ± 1.01	ns	0.345	16.86 ± 1.57	*	0.048	16.93 ± 1.31	*	0.075	16.54 ± 1.59	ns	0.292
	**Term**	13.91 ± 3.38			14.05 ± 3.71			14.75 ± 3.11			14.53 ± 2.68		
**Signal Transduction**	**Preterm**	1.56 ± 0.43	*	0.105	2.50 ± 0.49	*	0.066	2.44 ± 0.47	*	0.009	2.23 ± 0.47	ns	0.283
	**Term**	2.05 ± 0.71			1.89 ± 0.58			1.96 ± 0.31			1.93 ± 0.49		
**Signaling Molecules and Interaction**	**Preterm**	0.27 ± 0.07	*	0.147	0.16 ± 0.08	*	0.071	0.15 ± 0.06	*	0.039	0.17 ± 0.05	ns	0.353
	**Term**	0.22 ± 0.08			0.22 ± 0.08			0.21 ± 0.06			0.20 ± 0.07		

## Supplementary Materials

Supplementary materials can be found at http://www.mdpi.com/1422-0067/17/5/649/s1.
