# Supplementary material for: Impact of Prematurity and Perinatal Antibiotics on the Developing Intestinal Microbiota: A Functional Inference Study
Source: Int J Mol Sci. 2016 Apr 29;17(5):649. doi: 10.3390/ijms17050649 (PMC4881475; doi:10.3390/ijms17050649)
Supplement: Supplementary file 1 [file ijms-17-00649-s001.pdf]

# Supplementary Materials: Impact of Prematurity and Perinatal Antibiotics on the Developing Intestinal Microbiota: A Functional Inference Study

**Silvia Arboleya, Borja Sánchez, Gonzalo Solís, Nuria Fernández, Marta Suárez, Ana M. Hernández-Barranco, Christian Milani, Abelardo Margolles, Clara G. de los Reyes-Gavilán, Marco Ventura and Miguel Gueimonde**

**Table S1.** Median (Inter-quartile range) of the SCFA concentrations (µg/g faeces) in preterm infants according to the antibiotics use group. 1, Mother no antibiotics-Infant no antibiotic; 2, Mother no antibiotics-Infant antibiotics; 3, Mother antibiotics-Infants no antibiotics; and 4, Mother antibiotics-Infants antibiotics.

| SCFA | Antibiotic Use Group | Days of Life | | | |
|---|---|---|---|---|---|
| | | 2 Days | 10 Days | 30 Days | 90 Days |
| Acetate | 1 | 744.84 (734,77–928,71) | 1767.49 (1505.00–5095.13) | 3999.18 (3068.88–7530.40) | 3663.52 (3280.00–7773.39) |
| | 2 | 954.96 (867.30–1323.09) | 3279.62 (1628.99–6122.98) | 5310.76 (2896.19–8296.69) | 5412.50 (3895.97–6993.09) |
| | 3 | 768.58 (658.43–978.31) | 1651.27 (1294.98–1778.98) | 2459.88 (1634.87–4598.16) | 2944.74 (2658.58–4475.90) |
| | 4 | 878.05 (760.26–1268.58) | 1455.99 (1265.50–1724.09) | 2910.82 (2349.18–3659.05) | 4562.06 (3505.78–5740.70) |
| Propionate | 1 | 0.00 (0.00–349.07) | 360.90 (351.97–664.85) | 474.04 (369.71–865.15) | 1252.62 (935.21–1614.96) |
| | 2 | 338.02 (0.00–347.20) | 430.70 (86.22–677.95) | 447.63 (355.02–744.24) | 592.39 (386.63–1347.23) |
| | 3 | 0.00 (0.00–340.25) | 345.60 (0.00–362.50) | 454.81 (367.15–539.19) | 675.84 (597.73–738.66) |
| | 4 | 168.93 (0.00–359.52) | 350.40 (338.03–364.92) | 400.28 (350.70–500.68) | 1008.68 (695.77–1172.52) |
| Butyrate | 1 | 0.00 (0.00–0.00) | 0.00 (0.00–223.58) | 165.48 (0.00–399.53) | 379.65 (252.13–531.67) |
| | 2 | 0.00 (0.00–0.00) | 0.00 (0.00–0.00) | 221.64 (50.01–307.89) | 265.84 (39.52–494.74) |
| | 3 | 0.00 (0.00–118.56) | 0.00 (0.00–0.00) | 197.85 (82.92–282.42) | 252.54 (91.14–380.91) |
| | 4 | 0.00 (0.00–0.00) | 0.00 (0.00–0.00) | 0.00 (0.00–307.46) | 237.66 (76.89–874.39) |
| Total Acids | 1 | 744.84 (734.77–1355.08) | 2123.61 (1859.35–5983.55) | 4383.21 (4100.98–8260.43) | 6055.75 (4958.68–9048.68) |
| | 2 | 1298.35 (867.30–1667.60) | 3522.67 (1981.45–6934.11) | 6296.66 (3778.32–8767.26) | 6223.53 (4508.90–8803.88) |
| | 3 | 934.08 (658.43–1358.79) | 1996.87 (1294.98–2141.48) | 3161.97 (2330.86–5149.11) | 4126.79 (3434.84–5381.24) |
| | 4 | 1046.98 (760.26–1628.11) | 1809.29 (1630.23–2074.49) | 3709.85 (2697.00–4427.00) | 5420.75 (4526.06–7980.49) |
